# Case report: Innovative approach to esophageal cancer with right aortic arch: neoadjuvant immunotherapy and 3D reconstruction

**DOI:** 10.3389/fonc.2024.1496265

**Published:** 2025-01-13

**Authors:** Chengwen Luo, Zhilin Luo

**Affiliations:** Department of Thoracic Surgery, The Third Affiliated Hospital of Chongqing Medical University, Chongqing, China

**Keywords:** esophageal cancer, right aortic arch, immunotherapy, three-dimensional reconstruction, minimally invasive surgery

## Abstract

**Background:**

We report a rare case of locally advanced esophageal cancer with a right aortic arch (RAA), successfully treated with neoadjuvant immunotherapy and minimally invasive esophagectomy, guided by three-dimensional (3D) reconstruction.

**Case presentation:**

A 50-year-old male with stage III esophageal squamous cell carcinoma (cT3N0M0) and RAA underwent four cycles of neoadjuvant immunotherapy with sintilimab, resulting in significant tumor regression. Minimally invasive esophagectomy was performed with the aid of preoperative 3D reconstruction, which was critical in navigating the complex vascular anatomy and ensuring surgical precision.

**Conclusion:**

This case demonstrates the efficacy of neoadjuvant immunotherapy combined with 3D reconstruction in managing esophageal cancer complicated by vascular anomalies. This approach offers a promising alternative for complex cases where conventional treatments pose higher risks.

## Introduction

Esophageal cancer is a highly aggressive malignancy, ranking among the leading causes of cancer-related deaths globally (1). Despite advances in multimodal treatment approaches, including surgery, chemotherapy, and radiotherapy, the prognosis for locally advanced esophageal cancer remains poor (2). Moreover, anatomical variations such as right aortic arch (RAA), a rare congenital vascular anomaly, introduce significant complexities to surgical management, increasing the risks of complications and limiting treatment options.

While neoadjuvant chemotherapy and radiotherapy are standard strategies for improving resectability and survival in advanced esophageal cancer, recent advancements in immunotherapy have shown remarkable efficacy in enhancing antitumor immune responses. Immune checkpoint inhibitors, such as anti-PD-1 agents, have revolutionized cancer treatment by improving outcomes across various malignancies, including esophageal cancer (3, 4). However, there is limited evidence on the safety and efficacy of immunotherapy in patients with complex vascular anomalies like RAA, as the anatomical challenges may further complicate surgical interventions.

In parallel, the use of advanced surgical planning tools, such as three-dimensional (3D) reconstruction, has gained attention for its ability to provide detailed visualization of intricate anatomical structures, allowing for more precise and safer operations. The integration of 3D reconstruction with neoadjuvant immunotherapy presents an innovative approach, particularly in cases where conventional treatment modalities may pose increased risks due to anatomical anomalies.

In this report, we present a unique case of a patient with locally advanced esophageal squamous cell carcinoma and RAA who was successfully treated with neoadjuvant immunotherapy followed by minimally invasive esophagectomy guided by 3D reconstruction. This case not only underscores the potential of immunotherapy in complex surgical cases but also highlights the importance of tailored surgical planning in overcoming anatomical challenges. Our findings raise important considerations for expanding the use of these combined modalities in similarly complex oncological scenarios, warranting further investigation in larger clinical studies.

## Case presentation

**Figure 1:**
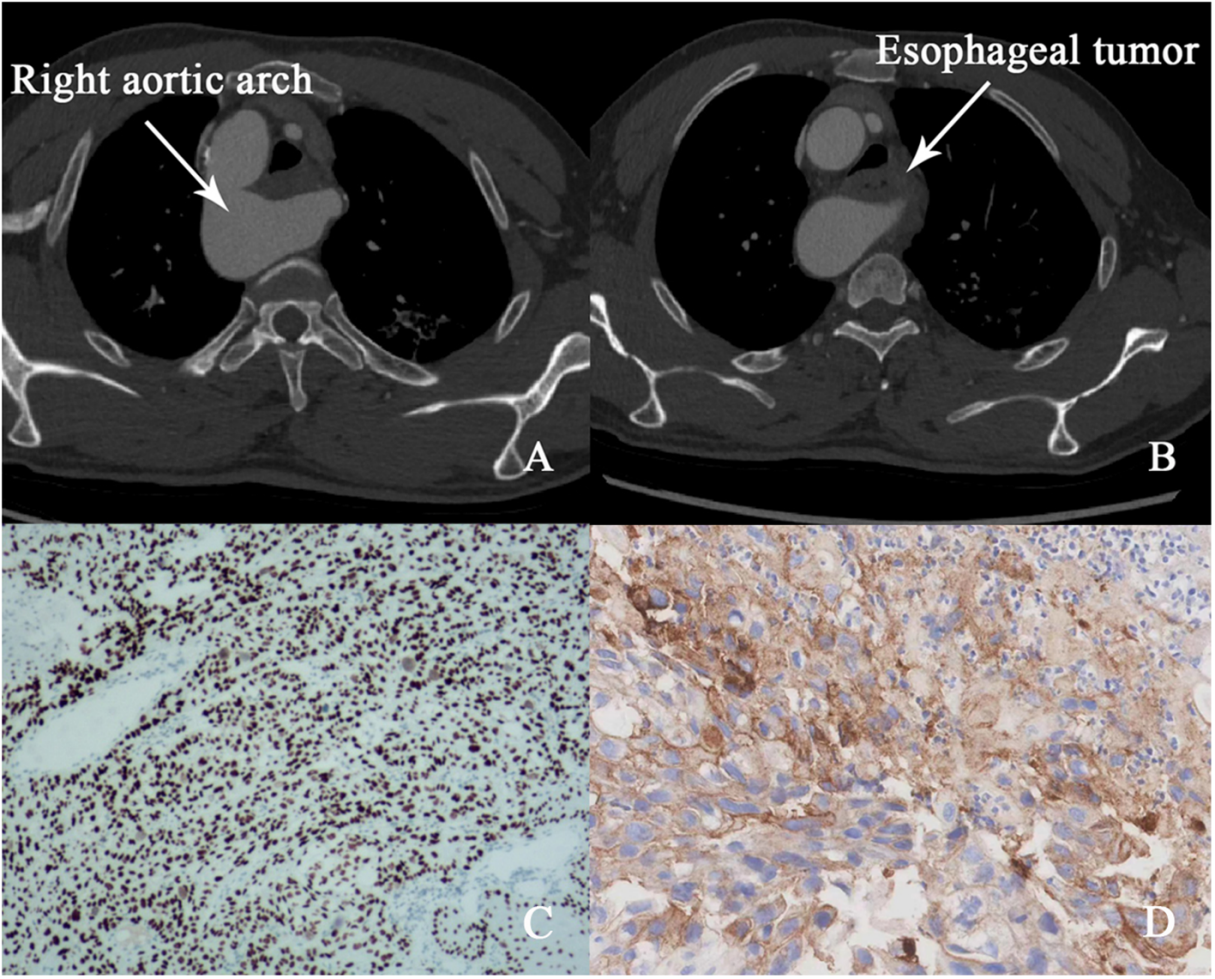
**(A, B)** Pre-treatment CT image (right aortic arch and esophageal tumor). **(C)** Microscopic image of esophageal squamous cell carcinoma. **(D)** PD-L1 expression results.

A 50-year-old male presented with progressive dysphagia over three months. Endoscopic evaluation revealed an esophageal mass 23-28cm from the incisors, with histopathology confirming esophageal squamous cell carcinoma (**Figure 1C**). Endoscopic ultrasound indicated tumor invasion into the esophageal adventitia, and staging computed tomography (CT) demonstrated a rare congenital vascular anomaly, right aortic arch (RAA), complicating the tumor’s location in the mid-upper thoracic esophagus (**Figures 1A, B**). No evidence of distant metastasis was found on magnetic resonance imaging (MRI) of the brain, bone scan, or abdominal CT. The clinical stage was determined to be cT_3_N_0_M_0_ (Stage III).

RAA, a rare vascular anomaly, posed significant challenges for surgical resection due to the abnormal positioning of major vessels relative to the esophagus. The presence of RAA increases the risk of intraoperative complications, such as vascular injury, and limits the feasibility of conventional surgical approaches. Given these anatomical complexities, a multidisciplinary team (MDT) convened to assess therapeutic options. Surgery was deemed high-risk due to the aberrant vasculature, and radiotherapy was excluded to avoid potential fibrotic changes that could further complicate resection. Considering the tumor’s high programmed death-ligand 1 (PD-L1) expression (CPS 50, **Figure 1D**), which is predictive of a favorable response to immunotherapy, the MDT elected to pursue neoadjuvant immunotherapy with the PD-1 inhibitor sintilimab (200 mg every three weeks).

**Figure 2:**
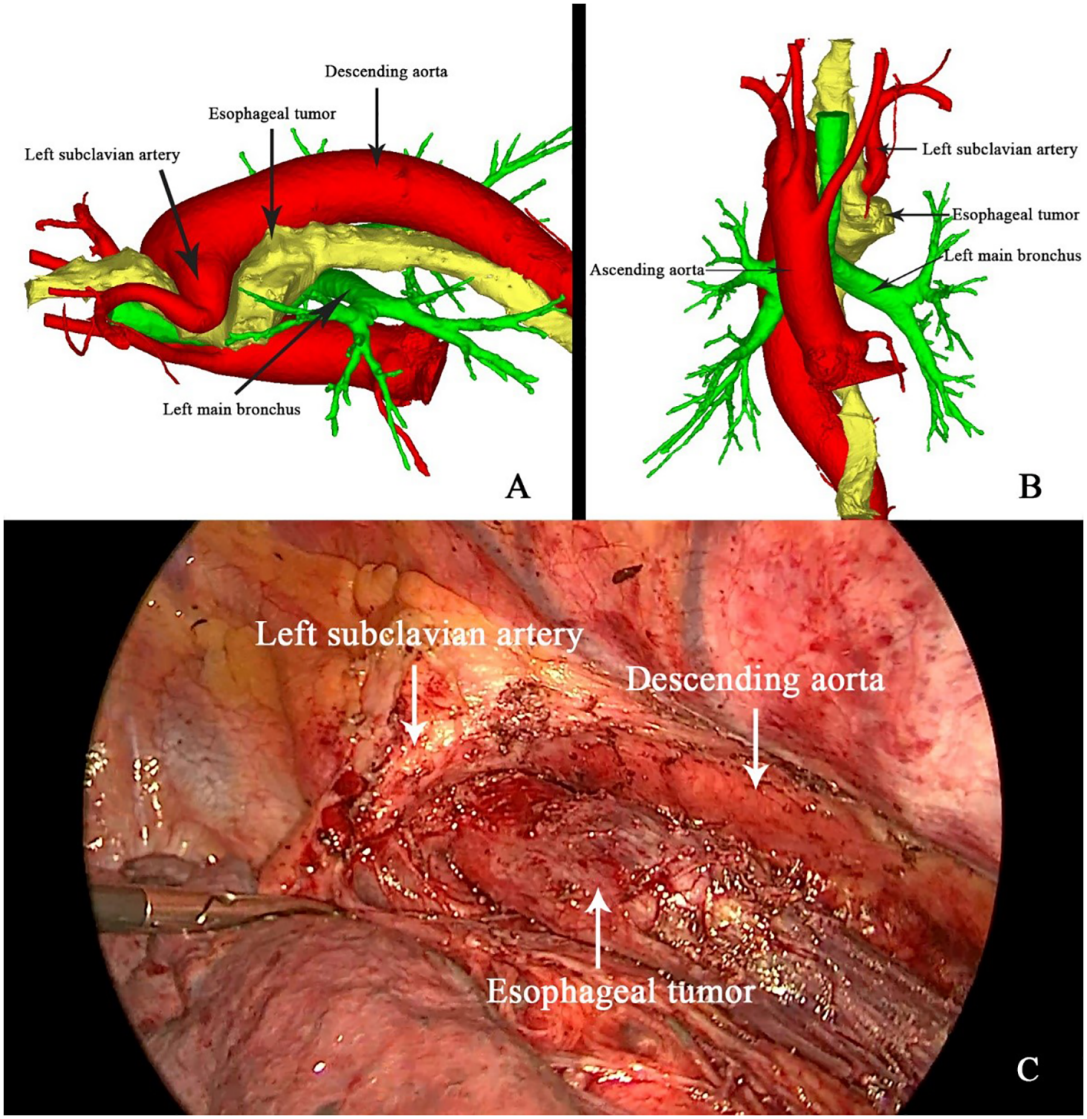
**(A)** Image of esophageal tumor under endoscopic ultrasound. **(B, C)** Comparison of esophageal tumor on CT images before and after neoadjuvant therapy.

After four cycles of immunotherapy, the patient’s dysphagia fully resolved, and he resumed a regular diet. Follow-up imaging, including CT and positron emission tomography-computed tomography (PET-CT), showed marked tumor regression with no signs of lymph node involvement or distant metastasis (**Figures 2A-C**). The significant tumor reduction and absence of metastasis indicated that surgical intervention was now feasible.

**Figure 3:**
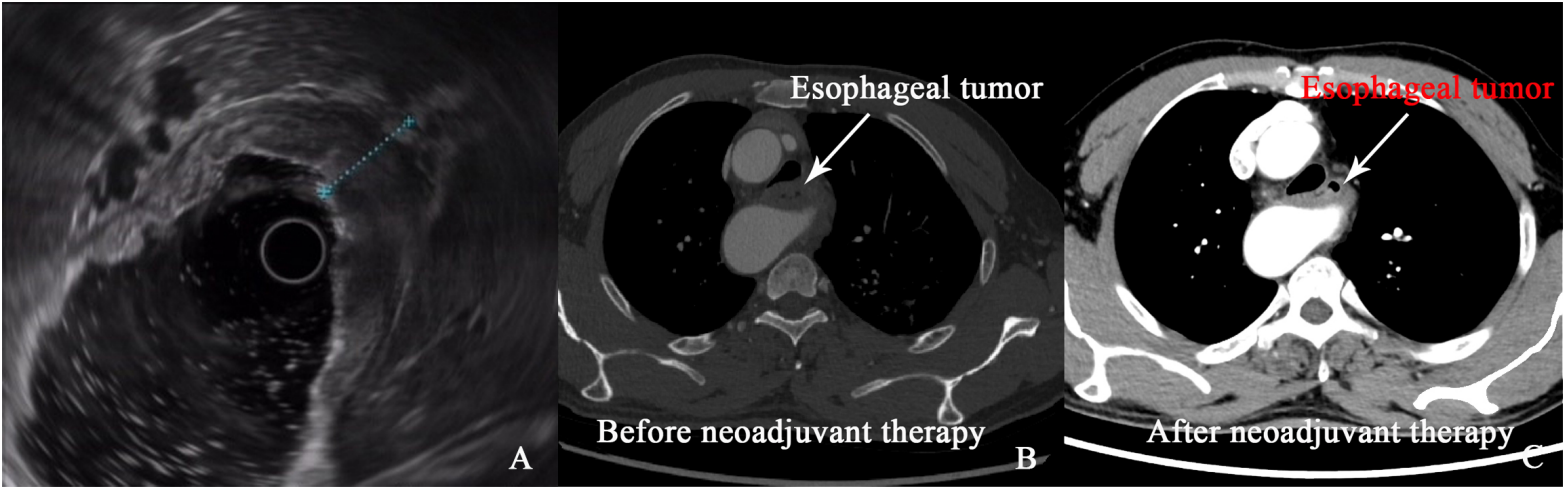
3D reconstruction **(A, B)** and intraoperative guidance **(C)** of the right aortic arch and esophageal tumor.

To mitigate the risks associated with the patient’s complex vascular anatomy, three-dimensional (3D) reconstruction was employed for preoperative planning. Thin-slice contrast-enhanced CT data were imported into the Mimics software (version 21, developed by Materialize, Belgium) to create a detailed 3D model of the esophagus and surrounding vasculature. This model allowed for precise visualization of the tumor’s proximity to the right aortic arch and the left subclavian artery, which crossed above the esophagus (**Figures 3A, B**). The 3D reconstruction was instrumental in guiding surgical planning, enabling the surgical team to anticipate and navigate the complex anatomical relationships intraoperatively, thereby minimizing the risk of vascular injury.

On April 26, 2020, the patient underwent minimally invasive thoracoabdominal esophagectomy with cervical anastomosis. Intraoperative findings confirmed the presence of the anomalous vascular anatomy, specifically the left subclavian artery originating from the right aortic arch and crossing anterior to the esophagus (**Figure 3C**). The operation proceeded with meticulous dissection around the esophagus and major vessels, with intraoperative guidance from the 3D reconstruction model. The esophageal tumor was completely resected, and mediastinal lymphadenectomy was performed without complication. Pathological examination revealed that the tumor had regressed to yp-T2N0M0 (Stage I), with no evidence of lymph node metastasis.

The patient’s postoperative recovery was uneventful, with no occurrences of anastomotic leakage, respiratory complications, or other major morbidities. He was discharged on postoperative day 8 in stable condition. Postoperative immunotherapy was resumed with sintilimab, following the same regimen as preoperatively, and was continued as maintenance therapy for two years. During the course of immunotherapy, no immune-related adverse events or complications were observed. At 48 months of follow-up, the patient remained disease-free with no evidence of recurrence or metastasis and reported a high quality of life.

## Discussion

### The role of neoadjuvant immunotherapy in complex anatomical cases

Neoadjuvant therapy has long been established as a critical component in the management of locally advanced esophageal cancer, traditionally involving chemotherapy or chemoradiotherapy to downstage tumors prior to surgery (5). In this report, we present a novel approach using neoadjuvant immunotherapy in a patient with esophageal squamous cell carcinoma (ESCC) complicated by a right aortic arch (RAA), an exceedingly rare vascular anomaly. The use of immune checkpoint inhibitors (ICIs), specifically anti-PD-1 agents such as sintilimab, has demonstrated considerable efficacy in various malignancies, including esophageal cancer (6). However, its application in cases complicated by anatomical anomalies like RAA has not been previously reported.

The mechanism by which immunotherapy aids in such complex anatomical cases may be related to its ability to enhance T-cell-mediated cytotoxicity against tumor cells, promoting both direct tumor regression and indirect reshaping of the tumor microenvironment (7). This reduction in tumor bulk not only decreases the physical space occupied by the malignancy but also potentially reduces inflammation and fibrosis around critical vascular structures, making them more accessible for surgical manipulation. In cases like ours, where radiotherapy could have induced fibrotic changes complicating surgical resection, the anti-fibrotic advantage of immunotherapy may represent a key factor in improving operative outcomes (8).

In this case, immunotherapy provided a safer alternative to chemoradiotherapy, particularly given the risk of fibrosis associated with radiotherapy, which could exacerbate surgical complexity. By enhancing the immune response against the tumor, immunotherapy allowed for significant tumor shrinkage without compromising the anatomical structures crucial for surgery. After four cycles of sintilimab, the tumor size was notably reduced, enabling a safer surgical approach in a challenging anatomical environment. This underscores the potential of immunotherapy not only in reducing tumor burden but also in minimizing perioperative complications in patients with complex vascular anomalies.

Following a systematic search of the PubMed database using the keywords “right aortic arch” and “esophageal cancer,” we identified a range of cases detailing the management of this rare condition. A review of the literature, as summarized in **Table 1** (9–22), shows that neoadjuvant chemoradiotherapy has been widely used in managing esophageal cancer, but immunotherapy remains underexplored in this context. This case suggests that ICIs may offer a promising neoadjuvant strategy in anatomically complex cases, providing similar benefits to traditional approaches but without the added risks associated with radiation-induced tissue changes.

**Table 1:** Literature review of treatment approaches for patients with esophageal cancer and right aortic arch.

Author	Year of Publication	Age	Gender	Tumor Type	Surgical Approach	Neoadjuvant Therapy	Neoadjuvant Treatment Plan	Follow-up Results
Yano et al. (9)	1998	52	Male	Esophageal squamous cell carcinoma	Left thoracotomy	Yes	Concurrent chemoradiotherapy	Not mentioned
Saito et al. (10)	1999	68	Male	Esophageal squamous cell carcinoma	Left thoracotomy + mediastinal lymphadenectomy	Yes	Neoadjuvant chemoradiotherapy	Died 13 months after surgery due to aortic rupture
Hanazono et al. (11)	2003	62	Male	Esophageal squamous cell carcinoma	Left thoracotomy	No	None	No recurrence
Shimakawa et al. (12)	2006	73	Male	Esophageal squamous cell carcinoma	Left thoracotomy	No	None	Not mentioned
Goto et al. (13)	2019	67	Male	Esophageal squamous cell carcinoma	Left thoracotomy	Yes	Neoadjuvant chemotherapy	Not mentioned
Kanaji et al. (14)	2013	52	Male	Esophageal squamous cell carcinoma	Left thoracotomy	No	None	Not mentioned
Kubo et al. (15)	2013	64	Male	Esophageal squamous cell carcinoma	Left thoracotomy	No	None	No recurrence
Linson et al. (16)	2017	73	Male	Esophageal adenocarcinoma	Right thoracoscopic esophagectomy	Yes	Neoadjuvant chemoradiotherapy	Complete pathological response, no recurrence
Okamura et al. (17)	2018	60	Female	Esophageal squamous cell carcinoma	Cervical and thoracoscopic surgery	No	None	No recurrence, good postoperative condition
Peng et al. (18)	2018	65	Male	Esophageal squamous cell carcinoma	Left thoracotomy	No	None	No recurrence
Ninomiya et al. (19)	2020	70	Male	Esophageal squamous cell carcinoma	Left thoracotomy	Yes	Neoadjuvant chemoradiotherapy	No recurrence, followed up for 22 months
Kumar et al. (20)	2020	62	Female	Esophageal squamous cell carcinoma	Right thoracoscopic esophagectomy	Yes	Neoadjuvant chemoradiotherapy	No recurrence
Nagano et al. (21)	2022	83	Male	Esophageal squamous cell carcinoma	Left thoracotomy	No	None	No recurrence, good postoperative condition
Hamada et al. (22)	2022	73	Male	Esophageal squamous cell carcinoma	Bilateral thoracoscopic resection	Yes	Neoadjuvant chemotherapy	No recurrence, discharged 26 days postoperatively

### The value of preoperative 3D reconstruction in surgical planning

Preoperative 3D reconstruction is an emerging tool in the surgical management of esophageal cancer, particularly in patients with anatomical anomalies such as RAA. In this case, 3D reconstruction provided critical insights into the spatial relationships between the tumor, the right aortic arch, and surrounding vascular structures, including the left subclavian artery. By allowing detailed visualization of the aberrant vasculature, 3D modeling enabled the surgical team to meticulously plan the operation, anticipate potential complications, and navigate around high-risk areas intraoperatively.

The ability to map out the patient’s vascular anatomy with such precision proved invaluable, especially in this case, where the aberrant vascular structures posed a significant risk for intraoperative vascular injury. Traditional imaging modalities, such as CT and MRI, can provide detailed cross-sectional images but lack the spatial integration needed to fully appreciate complex three-dimensional relationships. The use of 3D reconstruction in this case allowed for a more comprehensive understanding of the patient’s anatomy and significantly reduced the risks associated with the surgical resection.

Our review of the literature highlights the limited use of 3D reconstruction in cases of esophageal cancer complicated by RAA. Most previous cases relied on traditional imaging techniques, with few reports utilizing 3D technology to aid in surgical planning. This suggests an underutilization of a powerful tool that can enhance surgical safety and efficacy, particularly in anatomically challenging cases.

### Insights from the literature review

The table summarizing previous reports on esophageal cancer with RAA provides important insights into the current treatment landscape. It highlights a clear trend toward left thoracotomy as the preferred surgical approach due to the anatomical configuration of RAA, which complicates right-sided approaches. However, the table also reveals significant variability in the use of neoadjuvant therapies, with most cases relying on traditional chemoradiotherapy and none incorporating immunotherapy or 3D reconstruction.

This analysis underscores the novelty of our approach. By combining neoadjuvant immunotherapy with advanced 3D reconstruction, we were able to safely and effectively manage a highly complex case that would have otherwise posed significant challenges. The literature review further suggests that there is a need for greater exploration of these modern modalities in the treatment of esophageal cancer, particularly in patients with vascular anomalies. The use of 3D reconstruction in preoperative planning, combined with the immunomodulatory effects of checkpoint inhibitors, may represent a significant advancement in the treatment of these complex cases.

### Novelty and future directions

This case report is, to our knowledge, the first to combine neoadjuvant immunotherapy with 3D reconstruction in the treatment of esophageal cancer with RAA. The successful outcome in this case demonstrates the feasibility and potential benefits of this approach. Immunotherapy, by reducing tumor size without increasing surgical complexity, allowed for a safer and more effective resection. Meanwhile, 3D reconstruction provided critical preoperative insights that minimized intraoperative risks and improved surgical precision.

To further substantiate these findings, prospective studies examining the specific effects of immunotherapy on the surgical feasibility of anatomically complex cases are crucial. Such studies could assess perioperative outcomes, complication rates, and long-term survival compared to conventional chemoradiotherapy approaches. Additionally, exploring the biological and histopathological changes induced by immune checkpoint inhibitors in the tumor microenvironment of patients with vascular anomalies could offer deeper mechanistic insights. Multi-center clinical trials would also be beneficial in establishing standardized protocols for combining neoadjuvant immunotherapy with advanced surgical techniques like 3D reconstruction.

Future studies, including multi-institutional trials and larger case series, are needed to validate the effectiveness of this strategy and to explore its broader applicability. Additionally, the integration of other advanced technologies, such as artificial intelligence and augmented reality into 3D reconstruction, could further enhance its utility in complex surgical cases.

## Conclusion

This case highlights the successful use of neoadjuvant immunotherapy combined with preoperative 3D reconstruction in the management of esophageal cancer with RAA. This innovative approach offers a promising alternative to traditional treatment modalities, providing both oncologic efficacy and surgical safety in a challenging anatomical context. Our findings suggest that this combined modality should be further explored in future studies as a potentially superior strategy for managing esophageal cancer in patients with complex vascular anomalies.

## Data Availability

The original contributions presented in the study are included in the article/supplementary material. Further inquiries can be directed to the corresponding author.
